# Nucleoside transporter proteins as biomarkers of drug responsiveness and drug targets

**DOI:** 10.3389/fphar.2015.00013

**Published:** 2015-02-10

**Authors:** Marçal Pastor-Anglada, Sandra Pérez-Torras

**Affiliations:** ^1^Molecular Pharmacology and Experimental Therapeutics, Department of Biochemistry and Molecular Biology, Institute of Biomedicine, University of Barcelona, BarcelonaSpain; ^2^Oncology Program, CIBER ehd, National Biomedical Research Institute on Liver and Gastrointestinal Diseases, Instituto de Salud Carlos III, BarcelonaSpain

**Keywords:** nucleoside transporter, nucleoside analog, biomarker, cancer, ENT1, viral diseases

## Abstract

Nucleoside and nucleobase analogs are currently used in the treatment of solid tumors, lymphoproliferative diseases, viral infections such as hepatitis and AIDS, and some inflammatory diseases such as Crohn. Two gene families are implicated in the uptake of nucleosides and nucleoside analogs into cells, SCL28 and SLC29. The former encodes hCNT1, hCNT2, and hCNT3 proteins. They translocate nucleosides in a Na^+^ coupled manner with high affinity and some substrate selectivity, being hCNT1 and hCNT2 pyrimidine- and purine-preferring, respectively, and hCNT3 a broad selectivity transporter. SLC29 genes encode four members, being hENT1 and hENT2 the only two which are unequivocally implicated in the translocation of nucleosides and nucleobases (the latter mostly via hENT2) at the cell plasma membrane. Some nucleoside-derived drugs can also interact with and be translocated by members of the SLC22 gene family, particularly hOCT and hOAT proteins. Inter-individual differences in transporter function and perhaps, more importantly, altered expression associated with the disease itself might modulate the transporter profile of target cells, thereby determining drug bioavailability and action. Drug transporter pharmacology has been periodically reviewed. Thus, with this contribution we aim at providing a state-of-the-art overview of the clinical evidence generated so far supporting the concept that these membrane proteins can indeed be biomarkers suitable for diagnosis and/or prognosis. Last but not least, some of these transporter proteins can also be envisaged as drug targets, as long as they can show “transceptor” functions, in some cases related to their role as modulators of extracellular adenosine levels, thereby providing a functional link between P1 receptors and transporters.

Nucleosides and nucleotides play important roles in cell physiology both as nutrients and modulators of cellular homeostasis. They are implicated in crucial processes such as DNA and RNA synthesis, cell signaling, and metabolic regulation. Moreover, nucleoside and nucleobase analogs are currently used in the treatment of solid tumors, lymphoproliferative diseases, viral infections such as hepatitis and AIDS, and some inflammatory diseases such as Crohn (Minuesa et al., 2011; Jordheim et al., 2013). Nucleosides and nucleoside-derived drugs are hydrophilic molecules and diffuse, if they can, slowly across cell membranes. Thus, to exert their physiological and cytotoxic effects, specific membrane transporters that mediate their flux across cell membranes are required. Nucleoside transporters (NTs) are integral membrane proteins implicated in the salvage of natural nucleobases and nucleosides for nucleic acid synthesis. NTs belong to solute carrier families 28 and 29 (SLC28 and SLC29), which encode human concentrative nucleoside transporters (hCNT) and equilibrative nucleoside transporter proteins (hENTs), respectively (Molina-Arcas et al., 2009; Pastor-Anglada et al., 2009; Cano-Soldado and Pastor-Anglada, 2012; Molina-Arcas and Pastor-Anglada, 2013). However, the chemical modifications of the nucleoside analogs can alter their ability to interact with canonical transporter proteins implicated in the uptake of natural nucleosides. The paradigm of this concept is the lack of interaction of hCNT1 with those antiviral drugs lacking the 3^′^OH of the ribose, which appears to be an essential structural requirement for substrate translocation (Cano-Soldado et al., 2004). In fact, the structural requirements for nucleoside-NT interactions have already been reviewed elsewhere (Cano-Soldado and Pastor-Anglada, 2012). Therefore, in some cases additional carrier proteins become major players in drug bioavailability, and likely, drug action. In this sense, members of the SLC22 gene family, which encode human organic cation transporters, (hOCTs) and organic anion transporters (hOATs) are also implicated in the uptake of nucleoside-derived drugs (Minuesa et al., 2011). Moreover, although both hENT1 and hENT2 have been reported to translocate some nucleobases (Yao et al., 2011), other transporter proteins might contribute to the uptake of purine and pyrimidine nucleobases into cells despite the molecular entity behind them still have to be identified (Wallace et al., 2002; Quashie et al., 2010).

## NUCLEOSIDE-DERIVED DRUG TRANSPORTERS

### SLC28

The three human CNT proteins (hCNTs) mediate the unidirectional flow of nucleosides in an energy-costly process coupled to the influx of sodium ions. All three members of the SLC28 gene family accept uridine as substrate, but differ in their selectivity for other substrates. Thus, hCNT1 prefers pyrimidine nucleosides, hCNT2 purine nucleosides and uridine, and hCNT3 transports both pyrimidine and purine nucleosides. Importantly, they all have the ability of translocating selected nucleoside-derivatives currently used in anticancer and antiviral therapies, being major determinants of drug action (Huber-Ruano and Pastor-Anglada, 2009; Errasti-Murugarren and Pastor-Anglada, 2010). The sodium/nucleoside coupling ratio of hCNT1 and hCNT2 is 1:1, while hCNT3 shows a 2:1 stoichiometry (Smith et al., 2004, 2007). Interestingly, hCNT3 is the only transporter in this family able to accept protons as driving force (Smith et al., 2005; Gorraitz et al., 2010). Based upon its broad selectivity and highly potential concentrative activity, hCNT3 is considered to be a major player in nucleoside-derived drug uptake.

hCNT proteins, initially thought to be expressed almost exclusively in polarized epithelia, are in fact broadly expressed but not ubiquitous. In most polarized epithelia, these proteins localize at the apical membrane, thus facilitating vectorial flux of nucleosides across the barriers (Mangravite et al., 2001, 2003; Lai et al., 2002; Errasti-Murugarren et al., 2007). In fact, CNTs are expressed in all tissues which are considered to be relevant for drug pharmacokinetics (i.e., intestine, kidney, liver, and blood brain barrier).

### SLC29

The human ENT proteins (hENTs) family contain four members, hENT1-4 (Molina-Arcas et al., 2009; Pastor-Anglada et al., 2009; Cano-Soldado and Pastor-Anglada, 2012; Molina-Arcas and Pastor-Anglada, 2013). hENTs, except hENT4, mediate the facilitative transport of natural nucleosides with broad selectivity but relatively lower affinity than their CNT-type counterparts (Baldwin et al., 2004; Young et al., 2008). In fact, hENT1-3 proteins transport both purine and pyrimidine nucleosides, despite significant differences in substrate selectivity. The two best-characterized transporters hENT1 and hENT2 can be distinguished on the basis of their sensitivity to inhibition by the nucleoside analog NBTI, with hENT1 being much more sensitive than hENT2. Both transporters are also inhibited by vasodilation potentiators including dipyridamole and dilazep, being hENT1 also more sensitive than hENT2 (Visser et al., 2002). Additionally, hENT2 transports nucleobases (Osses et al., 1996). Moreover, hENT1 has recently been shown to transport some nucleobases, albeit with low kinetic efficiencies compared to hENT2 (Yao et al., 2011). hENT3 can also transport the nucleobase adenine and is not sensitive to inhibition by NBTI. This transporter protein was initially reported to be localized in lysosomes and, more recently, identified in mitochondria (Govindarajan et al., 2009). hENT3 is the only NT studied to date that has been associated with inherited human diseases. Several syndromes, including the H syndrome (Molho-Pessach et al., 2008, 2014; Bolze et al., 2012; Huber-Ruano et al., 2012) and the pigmented hypertrichosis with insulin dependent diabetes (PHID) syndrome (Spiegel et al., 2010) have been associated with mutations in the SLC29A3 gene. Finally, hENT4 may not be considered a “canonical” NT because it mostly transports organic cations, although it is a suitable adenosine transporter under acidic pH conditions (Barnes et al., 2006).

hENT1 and hENT2 are ubiquitously distributed, but differ in abundance among tissues and cell types (Baldwin et al., 2004). These transporters seem to be mainly, but not exclusively, localized at the basolateral side of polarized epithelial cells (Lai et al., 2002; Mangravite et al., 2003), contributing to the vectorial flux of nucleosides across these barriers.

### SLC22

The organic ion transporter family, SLC22, includes 22 transporters. Within the family, there are several subfamilies, which consist of members that cluster together based on sequence homology, including the OATs, the OCTs, and the OCTNs.

hOCT1, hOCT2, and hOCT3 are encoded by genes SLC22A1-3 (Koehler et al., 1997; Grundemann et al., 1998; Grundemann and Schomig, 2000). Transport of organic cations by hOCTs is electrogenic, sodium-independent, and bidirectional across the plasma membrane. The driving force is supplied by the electrochemical gradient of the transported organic cation. These proteins display broad substrate selectivity, transport positively charged compounds with a relative molecular mass below 500 and target both endogenous (hormones, neurotransmitters, creatinine, and others) and exogenous molecules (antiviral, antidiabetic, antiemetic, cytostatic among other drugs). hOCTs exhibit broad tissue distribution and are expressed in epithelial cells and neurons (Koepsell et al., 2003; Muller et al., 2005), whereas its occurrence in immune system cells has also been reported (Minuesa et al., 2008).

hOAT1, hOAT2, hOAT3, and hOAT4 are encoded by genes SLC22A6-8 and 11 (Rizwan and Burckhardt, 2007; VanWert et al., 2010). hOATs exchange extracellular against intracellular divalent organic anions. The concentration of the intracellular organic anion must be higher in the cytosol than outside the cell in order to drive the uptake of organic anions through OATs. This concentration difference is maintained by sodium-coupled anion transporters located at the same membrane as the respective OATs. These proteins accept a huge variety of chemically unrelated endogenous and exogenous organic anions including many drugs (Koepsell, 2013). OATs play a pivotal role in renal excretion of water-soluble, negatively charged organic compounds including endogenous waste products, numerous drugs, and drug metabolites. OATs are located at the plasma membrane of epithelial cells of proximal tubules, the site of efficient renal organic anion secretion. Selected OATs are present also outside the kidneys, e.g., in liver, placenta, nasal epithelium, and brain, where they serve special functions (VanWert et al., 2010; Burckhardt, 2012; Koepsell, 2013).

Although the SLC22 family include other members, those are the ones that are most likely to transport nucleoside analogs (Errasti-Murugarren and Pastor-Anglada, 2010; Minuesa et al., 2011; Koepsell, 2013).

## NUCLEOSIDE ANALOGS IN MEDICINAL CHEMISTRY

### NUCLEOSIDE ANALOGS IN CANCER TREATMENT

Nucleoside analogs were among the first chemotherapeutic agents used in the treatment of malignant diseases and today their activity is well established, showing a broad clinical use. The research on purines and purine analogs was recognized with the Nobel Prize in Physiology or Medicine in 1988. In the early 50’ Gertrude Elion and George Hitchings developed thioguanine and 6-mercaptopurine (6-MP) against leukemia. Later, they also developed, azathioprine, a drug that prevents rejection of transplanted organs and allopurinol which is used in the treatment of gout. Essentially, their research involved the rational development of a series of new drugs based upon the understanding of basic biochemical and physiological processes (Elion, 1989). (http://www.nobelprize.org/nobel_prizes/medicine/laureates/1988/press.html).

The anticancer nucleosides include analogs of pyrimidine and purine nucleosides (**Table 1**). The basic mechanism of action of most nucleoside analogs used in cancer treatment is similar (Parker, 2009). They get into cells where they are converted to analogs of cellular nucleotides (often the real active drugs) by enzymes of either the purine or the pyrimidine metabolic pathways. Nucleotide drugs can then be incorporated into nucleic acids and, in most cases, inhibit enzymes implicated in DNA synthesis, causing DNA damage and induction of apoptosis. Selective differences among them can also be found and they often rely upon their ability to interact with enzymes of the purine and pyrimidine salvage pathways.

**Table 1:** Nucleoside analogs used for cancer treatment.

Drug name	Therapeutic use	Analogous to	FDA approval	Identified uptake transporter	Reference
Mercaptopurine	Lymphoproliferative diseases	Purine	1953	hCNT3, hENT1, hENT2	Fotoohi et al. (2006), Yao et al. (2011)
Cytarabine	Lymphoproliferative diseases	Pyrimidine	1969	hCNT1, hENT1, hENT2	Smith et al. (2004); Clarke et al. (2006)
Fludarabine	Lymphoproliferative diseases	Purine	1991	hCNT2, hCNT3, hENT1, hENT2	King et al. (2006)
Pentostatin	Lymphoproliferative diseases	Purine	1991	hENT1, hENT2	Wiley et al. (1991)
Cladribine	Lymphoproliferative diseases	Purine	1993	hCNT2, hCNT3, hENT1, hENT2	King et al. (2006)
Azacitidine	Lymphoproliferative diseases	Pyrimidine	2004	hCNT1, hCNT2, hCNT3, hENT1, hENT2, hENT3, hENT4	Rius et al. (2009), Damaraju et al. (2012)
Clofarabine	Lymphoproliferative diseases	Purine	2004	hCNT2, hCNT3, hENT1, hENT2	King et al. (2006)
Nelarabine (AraG)^1^	Lymphoproliferative diseases	Purine	2005		
Decitabine	Lymphoproliferative diseases	Pyrimidine	2006	hENT1, hENT2	Damaraju et al. (2012), Arimany-Nardi et al. (2014)
Floxuridine	Solid tumors	Pyrimidine	1970	hCNT1, hCNT2, hCNT3	Lang et al. (2001), Smith et al. (2004), Hu et al. (2006)
Gemcitabine	Solid tumors	Pyrimidine	1996	hCNT1, hCNT3, hENT1, hENT2, hENT3	Mackey et al. (1999), Hu et al. (2006), Govindarajan et al. (2009)
Capecitabine (5-DFUR)^2^	Solid tumors	Pyrimidine	1998		
5-Fluorouracil	Solid tumors	Pyrimidine	1998	hENT1, hENT2, hOAT2	Kobayashi et al. (2005), Yao et al. (2011)

*^1^Nelarabine is a prodrug of AraG, which is translocated by hENT1 and hENT2 (Prus et al., 1990).*

*^2^Capecitabine is a prodrug of 5-DFUR, which is translocated by hCNT1, hCNT2, hCNT3, hENT1 and hENT2 (Lang et al., 2001; Mata et al., 2001; Molina-Arcas et al., 2006; Errasti-Murugarren et al., 2007). In gray, transporters for which weak substrate interaction has been reported.*

In general, purine nucleosides work almost exclusively against hematological malignancies, while pyrimidine analogs typically show efficacy against both blood cancers and solid tumors. NTs display different affinities for the analogs and their substrate selectivity has been comprehensively reviewed elsewhere. Thus, the tissue distribution of the transporters has a large impact on their therapeutic effect. As expected, purine-based nucleoside analogs such as fludarabine, cladribine, and clofarabine are substrates for hENT1, hENT2, hCNT3, and hCNT2 (Lang et al., 2001; Molina-Arcas et al., 2005; King et al., 2006; Owen et al., 2006; Errasti-Murugarren and Pastor-Anglada, 2010). Conversely, pyrimidine analogs such as gemcitabine, cytarabine, and azacytidine are transported by hCNT1 in addition to hENT1, hENT2, and hCNT3 (Mackey et al., 1999; Smith et al., 2004; Clarke et al., 2006; Endo et al., 2007; Errasti-Murugarren et al., 2007; Errasti-Murugarren and Pastor-Anglada, 2010; Arimany-Nardi et al., 2014). Nucleobases, such as 5-fluorouracil and 6-MP have also been reported to interact with hENT1 and hENT2 proteins, although their affinity constants are within the mM range (Yao et al., 2011).

### NUCLEOSIDE ANALOGS IN VIRAL DISEASES

During the past two decades, antiviral drugs have become crucial in the management of several viral infections, including HSV, HIV, HBV, HCV, and CMV infections. Prominent among these drugs are nucleoside derivatives(**Table 2**), which can act as potent antiviral agents owing to their ability to inhibit viral DNA polymerases and reverse transcriptases, which have key roles in the various viral life cycles. Antiviral nucleoside and nucleotide analogs are structurally more diverse than anticancer nucleoside analogs, as they consist of nucleosides, nucleotides, and acyclic nucleosides (De Clercq and Holy, 2005). Acyclic nucleosides have been approved for the treatment of various DNA virus infections (cidofovir), hepatitis B (adefovir), and AIDS (tenofovir).

**Table 2:** Nucleoside analogs used as antiviral agents.

Drug name	Therapeutic use	Analogous to	FDA approval	Identified uptake transporter	Reference
Ribavirin	HCV	Purine	1998	hCNT2, hCNT3, hENT1, hENT2	Jarvis et al. (1998), Patil et al. (1998), Hu et al. (2006), Govindarajan et al. (2008), Fukuchi et al. (2010)
Sofosbuvir	HCV	Purine	2013	hOATP1B1	Furihata et al. (2014)
Adefovir	HBV	Purine	2003	hOAT1, hOAT3	Cihlar et al. (1999, 2001), Uwai et al. (2007)
Entecavir	HBV	Purine	2004	hOAT1, hOAT3, hPEPT2	Xu et al. (2013, 2014)
Telbivudine	HBV	Pyrimidine	2006		
Lamivudine	HIV, HBV	Pyrimidine	1995	hENT3, hOCT1, hOCT2, hOCT3	Jung et al. (2008), Govindarajan et al. (2009), Minuesa et al. (2009)
Tenofovir	HIV, HBV	Purine	2001	hOAT1, hOAT3	Cihlar et al. (2001), Uwai et al. (2007)
Emtricitabine	HIV, HBV	Pyrimidine	2003	hOCT1, hOCT2, hOCT3	Minuesa et al. (2009)
Zidovudine	HIV	Pyrimidine	1987	hCNT1, hCNT3, hENT2, hENT3, hOAT1, hOAT2, hOAT3, hOAT4	Yao et al. (2001), Takeda et al. (2002), Cano-Soldado et al. (2004), Smith et al. (2004), Baldwin et al. (2005), Hu et al. (2006), Minuesa et al. (2009)
Didanosine	HIV	Purine	1991	hCNT2, hCNT3, hENT1, hENT2, hENT3	Ritzel et al. (1998, 2001), Yao et al. (2001), Baldwin et al. (2005), Hu et al. (2006)
Zalcitabine	HIV	Pyrimidine	1992	hCNT3, hENT1, hENT2, hOCT1, hOCT2	Ritzel et al. (2001), Yao et al. (2001), Baldwin et al. (2005), Jung et al. (2008)
Stavudine	HIV	Pyrimidine	1994	hCNT1, hCNT3, hENT3	Cano-Soldado et al. (2004), Hu et al. (2006), Govindarajan et al. (2009)
Abacavir	HIV	Purine	1998	hOCT1, hOCT2, hOCT3	Minuesa et al. (2009)
Acyclovir	Herpes	Purine	1982	hOCT1, hOAT1, hOAT2	Takeda et al. (2002), Cheng et al. (2012)
Penciclovir	Herpes	Purine	2002	hOAT1, hOAT2, hOAT3	Cheng et al. (2012)
Famciclovir (Penciclovir)^1^	Herpes	Purine	2007		
Ganciclovir	Herpes, CMV	Purine	1989	hOCT1, hOAT1, hOAT2	Takeda et al. (2002), Cheng et al. (2012)
Valaciclovir (Acyclovir valyl ester)	Herpes, CMV, EBV	Purine	1996	hPEPT1, hPEPT2, ATB(0,+)	Ganapathy et al. (1998), Hatanaka et al. (2004)
Cidofovir	CMV	Pyrimidine	1996	hOAT1, hOAT3	Cihlar et al. (1999), Uwai et al. (2007)
Valganciclovir (Ganciclovir valyl ester)	CMV	Purine	2001	hPEPT1, hPEPT2, ATB(0,+)	Sugawara et al. (2000), Umapathy et al. (2004)

*^1^Famciclovir is a prodrug of Penciclovir.*

*In gray, transporters where only interaction has been demonstrated.*

Some NT proteins and particularly, but not exclusively, the broad selective transporter hCNT3 can interact and translocate different types of antiviral drugs, such as ribavirin and zidovudine, among others (Hu et al., 2006; Errasti-Murugarren et al., 2007). However, hOCT proteins also appear to interact with these drugs with high affinity, lamivudine being a substrate and tenofovir, abacabir, and zidovudine high affinity inhibitors of hOCT1, hOCT2, and hOCT3 (Minuesa et al., 2009). This might be particularly relevant in the clinics, because AIDS patients are under combined therapy and hOCT1 and hOCT3 are highly expressed in target cells (i.e., CD4^+^ T cells; Minuesa et al., 2008). Moreover, hOCTs (particularly hOCT1) are highly polymorphic proteins, with some variants showing high allelic frequency and already shown to be relevant in the pharmacokinetics of other drugs such as metformin (Errasti-Murugarren and Pastor-Anglada, 2010). Other SLC22 members might also interact with nucleoside-derived drugs (Errasti-Murugarren and Pastor-Anglada, 2010; Cano-Soldado and Pastor-Anglada, 2012), whereas for novel drugs at the pre-clinical stages, or even in the clinics such as the pronucleotide sofosbuvir, the best candidates to mediate their cellular uptake still have to be identified.

### NUCLEOSIDE ANALOGS IN INFLAMMATORY DISEASES

The thiopurines, 6-MP, and its pro-drug azathioprine (AZA) have been a cornerstone of medical management of patients with inflammatory bowel disease (IBD) and many rheumatoid disorders.

Thiopurines are metabolized to their end products, 6-methymercaptopurine (6-MMP) and the 6-thioguanine nucleotides (6-TGN). Although these nucleotides disturb proper DNA synthesis it has not been conclusively shown, that 6-TGN are the only molecules responsible for proper action. However, immunosuppressive function seems to be mediated in part by induction of apoptosis in lymphocytes. A correlation of therapeutic benefit and 6-TGN levels has been put into question. Although thiopurines are widely used, several safety concerns remain regarding the optimal treatment regimens. Thioguanine has been proposed as an alternative to overcome such problems, as it skips the metabolic conversion to 6-MMP which is responsible for hepatotoxicity (Bar et al., 2013; Frei et al., 2013; Friedman et al., 2014).

## TRANSPORTERS AS BIOMARKERS

Transporter function may be influenced by multiple factors and is likely to be highly variable among individuals (Nies et al., 2009; Sakamoto et al., 2013; Prasad et al., 2014). Thus, interindividual heterogeneity in response to therapy can be somehow related to inherent transporter function variability among patients and also to altered transporter expression in target cells, even as a result of the disease itself. In this context, transportome profiles and their associated pharmacogenetics might prove suitable for the prediction of treatment outcomes, and ideally should be helpful in decision making processes, such as choice of treatment (drug and dose) and in anticipating drug–drug interactions when patients face drug combination schedules. In fact, drug transporters can often be inhibited by numerous compounds, either other drugs or endogenous substrates, typically by competition for recognition and binding. These interactions may even result in altered drug pharmacokinetics. Genetic heterogeneity of the transporter-encoding genes may also determine variable transporter function, either by increasing or by reducing the individual overall exposure to a substrate, also depending upon the tissue expression and localization of particular transporters.

In summary, inter-individual differences in transporter expression and function and, perhaps more importantly, altered expression either due to the oncogenic process itself, to viral infection or to inflammation might modulate the transporter profile of target cells, thereby determining drug bioavailability and action.

### BIOMARKERS OF DRUG SUSCEPTIBILITY

As mentioned above, NT proteins are necessary for nucleoside analogs to enter into cells and exert their pharmacological action. The analysis of the role played by NTs in drug sensitivity and clinical outcomes of cancer patients initially focused on lymphoproliferative diseases.

Leukemic cells express both CNTs and hENTs and the analysis of these proteins in cells from 22 patients with primary CLL cells similarly expressed hENT1, hENT2, hCNT2, and hCNT3 mRNAs. However, fludarabine accumulation in CLL cells is mainly mediated by hENT1 and hENT2 (Molina-Arcas et al., 2003). Moreover, fludarabine transport correlates with hENT2 protein expression and activity, whereas in Mantle Cell Lymphoma (MCL) hENT1 correlates with *ex vivo* gemcitabine sensitivity (Marce et al., 2006).

Furthermore, data suggest that hENT1 expression influences response to cytarabine, but not sufficient to support its use as a biomarker for guiding treatment in Acute Myeloid Leukemia (AML). In any case, permeant nucleoside analogs have been designed as a way to bypass NT function. Thus the activity of elacytarabine (the elaidic acid ester derivative of cytarabine) is not significantly predicted by the hENT1 expression level (Rizzieri et al., 2014).

To date, most studies in solid tumors focused on hENT1 levels and clinical response to gemcitabine, mainly in pancreatic cancer (**Table 3**). Comprehensive *in vitro* evidence supports the view that hENT1 activity is a key determinant of gemcitabine action. Overexpression of hENT1 enhances gemcitabine response in human pancreatic cancer, and cells lacking hENT1 expression are highly resistant to gemcitabine (Mori et al., 2007; Perez-Torras et al., 2008, 2013). There is increasing evidence supporting the view that hENT1 is a predictive biomarker for the use of gemcitabine. Several studies have explored hENT1 expression in gemcitabine-treated solid tumors with various techniques (immunohistochemistry and qRT-PCR) and different treatment regimens trying to establish correlations with patient outcomes. The first studies demonstrated that hENT1 expression was prognostic in pancreatic cancer, but this research included both early (resected) and advanced disease, which were mostly treated with gemcitabine (Spratlin et al., 2004; Giovannetti et al., 2006). Later, in patients with pancreatic cancer from a randomized phase III RTOG 9704 study, hENT1 expression was associated with increased OS and DFS in patients who received adjuvant gemcitabine chemotherapy, but not on those who received 5-fluorouracil (Farrell et al., 2009). Moreover, in an analysis of 45 pancreatic cancer patients treated with postoperative adjuvant gemcitabine-based chemoradiation therapy, patients with high hENT1 expression had significantly longer DFS and OS than those with low hENT1 expression, and hENT1 expression was the only independent predictor for DFS and OS (Maréchal et al., 2009). Additional studies conducted up to now have provided encouraging but not yet convincing evidence of the use of this transporter as a suitable biomarker (Fujita et al., 2010; Kim et al., 2011; Kawada et al., 2012; Kondo et al., 2012; Maréchal et al., 2012; Morinaga et al., 2012; Murata et al., 2012; Fisher et al., 2013b; Nakagawa et al., 2013; Xiao et al., 2013). This is probably because they have been mostly retrospective and have used non-randomized series of patients. On the contrary, only a few studies have provided no evidence supporting the use of hENT1 as a predictive biomarker for gemcitabine efficacy in a neoadjuvant gemcitabine-based chemoradiation setting (Kawada et al., 2012) and in patients with advanced pancreatic cancer (Ormanns et al., 2014). However, none of the latter studies truly tested gemcitabine monotherapy in a defined group of patients. Recently, hENT1 expression was determined in a large unbiased group of patients that were given gemcitabine monotherapy (ESPAC3 trial), confirming its role as a predictive marker in gemcitabine-treated but not 5-fluorouracil-treated patients. This suggests that gemcitabine should not be used for patients with low tumor hENT1 expression (Greenhalf et al., 2014). Nevertheless, it cannot be ruled out the fact that differences among studies should also be explained by the tools used in this type of analysis. In this sense, it has been recently stated that the two antibodies used to detect hENT1 are not equivalent (Svrcek et al., 2014).

**Table 3:** hENT1 expression and clinical response.

Transporter	Treatment	Assay	Cancer type	*n* =	Analysis	Reference
hENT1	GE containing chemotherapy	IHQ	NSCLC	24	Response to therapy	Oguri et al. (2007)
hENT1	GE	qRT-PCR	Bladder	12	Complete response	Mey et al. (2006)
hENT1	GE-CDDP	IHQ	Bladder	40	OS	Matsumura et al. (2011)
hENT1	GE	IHQ	Biliary tract	31	TTP	Santini et al. (2011)
hENT1	GE	IHQ	Biliary tract	26	OS and PFS	Borbath et al. (2012)
hENT1	GE	IHQ	Biliary tract	105	OS	Kobayashi et al. (2012)
hENT1	GE	IHQ	Biliary tract	28	OS	Murata et al. (2013)
hENT1	GE monotherapy after operation	IHQ	Pancreas	434	OS	Greenhalf et al. (2014)
hENT1	GE	IHQ	Pancreas	169	Not a prediction biomarker	Ormanns et al. (2014)
hENT1	GE plus S-1 after operation	IHQ	Pancreas	109	OS and DFS	Nakagawa et al. (2013)
hENT1	GE-based chemoradiation after operation	IHQ	Pancreas	95	RFS	Fisher et al. (2013b)
hENT1	GE-based chemotherapy	IHQ	Pancreas	44	OS and DFS	Xiao et al. (2013)
hENT1	GE-based chemotherapy before operation	IHQ	Pancreas	55	OS	Murata et al. (2012)
hENT1	GE-based chemotherapy after operation	IHQ	Pancreas	27	OS and DFS	Morinaga et al. (2012)
hENT1	GE-based chemoradiation after operation	IHQ	Pancreas	222	OS	Maréchal et al. (2012)
hENT1	GE plus S-1	IHQ	Pancreas	86	OS and DFS	Kondo et al. (2012)
hENT1	GE-based chemotherapy after operation	qRT-PCR	Pancreas	40	OS	Fujita et al. (2010)
hENT1	GE-based chemoradiation after operation	IHQ	Pancreas	45	OS and DFS	Maréchal et al. (2009)
hENT1	GE-based chemoradiation after operation	IHQ	Pancreas	91	OS and DFS	Farrell et al. (2009)
hENT1	GE adjuvant and palliative	qRT-PCR	Pancreas	81	OS	Giovannetti et al. (2006)
hENT1	GE palliative	IHQ	Pancreas	21	OS	Spratlin et al. (2004)

Interestingly, correlations between intratumoral hENT1 expression and responsiveness to gemcitabine have also been reported to occur in other types of malignant tumors. Responses to gemcitabine-based chemotherapy were evident in patients with high hENT1 expression but not in patients without hENT1 expression in an analysis of 24 patients with non-small cell lung cancer (Oguri et al., 2007). In bladder cancer, a first study with 12 cancer specimens showed a significant correlation between gemcitabine chemotherapy outcome and hENT1 expression (Mey et al., 2006). Later, the OS of patients treated with a gemcitabine-/cisplatin-based combination chemotherapy was significantly higher in patients with high hENT1 expression (Matsumura et al., 2011). In addition, a few studies in biliary cancer have shown an association between hENT1 expression, as determined by immunohistochemistry, and chemosensitivity to gemcitabine (Santini et al., 2011; Borbath et al., 2012; Kobayashi et al., 2012; Murata et al., 2013).

### BIOMARKER OF PROGNOSIS

Besides the role of NT proteins as biomarkers of drug susceptibility discussed above, NT expression by itself might be a biomarker of disease prognosis, although available data are controversial so far. This is due to the fact that almost all studies have included treated-patients. Indeed, hENT1 itself has been related to epithelial mesenchymal transition (EMT) probably independently of its role as a drug transporter (Guillen-Gomez et al., 2012; Lee et al., 2014). Thus, it is likely that these proteins can also serve as prognostic biomarkers. Although several studies analyzed the prognostic value of intratumoral hENT1 expression in patients who had not received gemcitabine chemotherapy, in many of them it is not possible to discriminate between a therapeutic predictive effect and a disease prognostic effect for hENT1 expression due to the patient cohort choice, often based on retrospective analysis. A retrospective analysis of 111 patients with resected gastric cancer who had not received gemcitabine-based chemotherapy revealed that patients with high hENT1 expression had a shorter OS or DFS than those with low hENT1 expression (Santini et al., 2010). Similar results were also reported in 41 patients with ampullary cancer (Santini et al., 2008). In ampullary carcinoma, hENT1 expression and proliferation index were found to be dependent on the histological subtypes, suggesting a key role of hENT1 in tumor growth (Perrone et al., 2010). In biliary tract malignancies, high expression of hENT1 was correlated with improved OS, although the study was performed in 63 patients including intrahepatic, hilar, or distal cholangiocarcinoma and gallbladder carcinoma and a small group of patients within this cohort had been treated with nucleoside analogs (Fisher et al., 2013a). In addition, in a study with 84 pancreatic cancer patients, low expression of hENT1 was associated with shorter OS and progression free survival (PFS) independently of gemcitabine treatment (Kim et al., 2011). In an attempt to clarify this point, Greenhalf et al. (2014) examined the expression of hENT1 combining the observation arms from the ESPAC1 and ESPAC3 trials that were specifically randomized to no adjuvant treatment. Although the resultant number of cases investigated was small, there was no evidence to support hENT1 expression levels *per se* as indicative of OS (Greenhalf et al., 2014). However, further studies on a larger number of patients with various cancers are needed to clarify the role of hENT1 as a prognostic biomarker. Furthermore, the analysis of NTs expression in nearly 300 gynecologic tumors (ovary, endometrium, and uterine cervix carcinomas) showed loss of hCNT1 in a much higher number of cases than hENT1 and hENT2 and, this loss highly correlated with poor prognosis histotypes (Farre et al., 2004). In breast cancer, hCNT1 alone exhibited prognostic value for DFS and risk of relapse (Gloeckner-Hofmann et al., 2006).

A major bottleneck in the interpretation of the available data is the fact that almost all the studies addressed to determine the role of NTs as prognosis biomarkers in cancer analyzed different grades of the disease without a direct comparison with healthy tissue.

### GENETIC HETEROGENEITY AS BIOMARKER

Compared to other drug transporter encoding-genes, neither SLC28 nor SLC29 genes appear to be highly polymorphic in humans (Errasti-Murugarren and Pastor-Anglada, 2010). On the contrary, hOCTs are highly polymorphic proteins, with some variants showing high allelic frequency in humans, being relevant to the pharmacokinetics of selected drugs such as metformin (Errasti-Murugarren and Pastor-Anglada, 2010).

Pharmacogenetic studies of hENT1 have not clearly identified yet any clinical relevance of the inter-individual sequence variations in the hENT1 encoding gene (SLC29A1). Although single nucleotide polymorphisms (SNPs) in hENT1 have been identified, none have demonstrated functional consequences in terms of drug uptake or accumulation (Osato et al., 2003; Kim et al., 2006; Myers et al., 2006). However, a recent study of 154 patients treated with neoadjuvant gemcitabine suggested that a combined assessment of six SNPs, including the hENT1 T-549C allele and hENT1 C913T allele, did correlate to OS (Okazaki et al., 2010). While multiple alternatively spliced variants encoding hENT1 have been identified, they have not been shown to have clinical relevance so far. As for hENT1, hENT2 also shows different spliced variants, some of them likely to be translated into proteins, although their physiological relevance is also unknown (Mangravite et al., 2003). Regarding the other members of the SLC29 gene family, as mentioned above, hENT3 has been directly implicated in the pathogenesis of human disease. Loss-of-function mutations in the hENT3 encoding gene have been associated with familial Rosai-Dorfman disease, Faisalabad histiocytosis, H syndrome, and PHID (Molho-Pessach et al., 2008; Cliffe et al., 2009; Kang et al., 2010; Morgan et al., 2010; Spiegel et al., 2010; Avitan-Hersh et al., 2011). Despite some controversy about the intracellular location of this transporter protein, it has recently been shown that ENT3 null mice develop defects in the lysosomal system, causing ineffective apoptotic cell clearance and increased M-CSF signaling which contribute to increase macrophage number and histiocytosis (Hsu et al., 2012).

Although hCNTs do not appear to be particularly variable at the gene level, some polymorphisms have been identified and supposed to have clinical relevance (Errasti-Murugarren and Pastor-Anglada, 2010). Functional complexity of selected polymorphic variants can be paradigmatic, as for the Spanish hCNT3 variant (hCNT3 Cys602Arg) which shows variable affinities for hCNT3 substrates, apparent loss of interaction with one of the two Na^+^ ions being translocated along with the drug, and lipid raft missorting of the mutated variant, thereby dramatically affecting its biological function (Errasti-Murugarren et al., 2008, 2010; Cano-Soldado et al., 2012; Cano-Soldado and Pastor-Anglada, 2012). Genetic variability of the SLC28 genes has recently been addressed also in the context of ribavirin-based therapies in patients infected with the HCV. The analysis of SLC28A2, SLC28A3, SLC29A1, and SLC29A2 variants in a cohort of 169 patients cronically infected with HCV did not show any significant correlation with response to treatment. However, the SLC28A3 haplotype rs10868138G/rs56350726T was associated with protection against ribavirin-induced hemolytic anemia (Doehring et al., 2011), a finding which may be difficult to interpret as long as hENT1 is the major NT in erythrocytes. Moreover, the SLC28A3 rs56350726T variant has also been associated with SVR in a cohort of 216 patients. Some of these polymorphisms might probably affect ribavirin pharmacokinetics, as for the SLC28A2 rs11854484 variant, which was linked in the same study to high plasma ribavirin levels during combined PegIFN-α/ribavirin treatment (Rau et al., 2013). The same SLC28A2 variant was previously significantly associated with SVR (D’Avolio et al., 2012). Interestingly, the SCL28A2 gene product, hCNT2, has recently been shown to be a ribavirin transporter that is regulated by INF-α (Pinilla-Macua et al., 2014). Notwithstanding, considering the impact of ribavirin transporters in the clinical outcome there are limited data relative to the expression of these transporters and their polymorphisms.

## NTs AS DRUG TARGETS

Nucleotides and nucleosides can be supplied by either salvage or by de novo synthesis from smaller precursors. NTs perform a crucial role in maintaining nucleoside homeostasis under physiological conditions through provision of nucleosides and nucleobases derived from the diet or produced by tissues such as liver. In this sense, as mentioned above NTs show less functional diversity than other transporter proteins and it has been suggested, as for hCNT3 as a paradigm within this family, that any change can critically compromise “fitness” in humans (Badagnani et al., 2005). This would be consistent with low evolutionary-related heterogeneity and, at least up to now, no genetic-based diseases associated with this gene family.

### MORE THAN TRANSPORTERS

For years the study of these membrane proteins in the context of anticancer therapy has focused on the role these proteins might play in drug efficacy and safety. Notwithstanding, cancer represents an important pathophysiological condition that requires abnormally high levels of nucleoside influx to support higher amounts of DNA synthesis associated with the disease. Most cells express several NT-encoding genes, thereby anticipating some sort of apparent functional redundancy when analyzing transporter profiles, particularly considering that transporters often show overlapping or even identical selectivity profiles. Indeed, hCNT expression is commonly associated with fully differentiated cell types (Molina-Arcas et al., 2009; Pastor-Anglada et al., 2009; Cano-Soldado and Pastor-Anglada, 2012; Molina-Arcas and Pastor-Anglada, 2013) and oncogenesis often results in hCNT down-regulation, particularly in hCNT1 (Farre et al., 2004; Zollner et al., 2005; Lane et al., 2010; Bhutia et al., 2011; Martinez-Becerra et al., 2012; Mohelnikova-Duchonova et al., 2013; **Table 4**). However, the evidence of hCNT1 loss during oncogenesis needs further investigation, because almost all the studies performed so far focused exclusively on hCNT1-related mRNA levels. We have recently demonstrated that restoration of hCNT1 function in pancreatic adenocarcinoma cell lines is able to induce cell cycle arrest, increase cell death by a non-apoptotic mechanism, trigger changes in some intracellular signaling cascades and inhibit cell migration (Perez-Torras et al., 2013). More importantly, all these events can also be induced when expressing a mutated hCNT1 protein that localizes to the plasma membrane but lacks the ability to translocate substrates. Remarkably, hCNT1 protein restoration can also inhibit tumor growth in a mouse model of pancreatic adenocarcinoma (Perez-Torras et al., 2013). These observations would argue in favor of hCNT1 being a transceptor protein. This new concept within the field comes from seminal studies both in mammalian cells and yeast: a transporter that in a substrate translocation-dependent or -independent manner is able to modulate cell functions, behaving as a signaling-inducer molecule or a receptor itself. Several examples of SLC membrane transporters with these additional regulatory roles have recently been reported in the literature (Lacoste et al., 2012; Coothankandaswamy et al., 2013; Perez-Torras et al., 2013; Tanaka et al., 2014).

**Table 4:** NTs expression in tumor tissue.

Transporter	Tumor	Assay	*n* =	Expression	Compared vs. healthy tissue	Reference
hCNT1	Breast	IHQ		Decreased	Yes	Lane et al. (2010)
hCNT1	Pancreas	qRT-PCR, WB	5	Decreased	Yes	Bhutia et al. (2011)
hCNT1	Ovary	IHQ	90	Decreased	No	Farre et al. (2004)
hCNT1	Endometrium	IHQ	79	Decreased	No	Farre et al. (2004)
hCNT1	Uterine cervix	IHQ	118	Decreased	No	Farre et al. (2004)
hCNT1	Pancreas	qRT-PCR	32	Decreased	Yes	Mohelnikova-Duchonova et al. (2013)
hCNT1	Hepatocarcinoma	qRT-PCR	10	Decreased	Yes	Zollner et al. (2005)
hCNT1	Bladder	qRT-PCR	12	Decreased	No	Mey et al. (2006)
hCNT3	Pancreas	qRT-PCR	32	Decreased	Yes	Mohelnikova-Duchonova et al. (2013)
hENT1	Breast	IHQ		Decreased	Yes	Lane et al. (2010)
hENT1	Pancreas	qRT-PCR	32	Decreased	Yes	Mohelnikova-Duchonova et al. (2013)

The possibility of hCNT proteins behaving as putative transceptors might also apply to the other two members of the *SLC28* gene family, hCNT2 and hCNT3. In contrast to hCNT1, both are high affinity adenosine transporters likely to modulate extracellular adenosine levels and, consequently, purinergic signaling. CNT2 was shown to be under the control of A1 adenosine receptors in hepatocytes by a mechanism which is dependent upon ATP-sensitive potassium channels and glucose availability (Duflot et al., 2004; Medina-Pulido et al., 2013). A similar receptor-transporter crosstalk has recently been reported for CNT2 in neurons (Medina-Pulido et al., 2013), being CNT2 expression down-regulated in the rat brain by sleep deprivation and experimental ictus (Guillen-Gomez et al., 2004; Medina-Pulido et al., 2013). Moreover, adenosine taken up by CNT2 is responsible for AMP-dependent protein kinase (AMPK) activation in epithelial and neuronal models (Aymerich et al., 2006; Medina-Pulido et al., 2013). As for CNT1, CNT2 expression appears to be characteristic of differentiated hepatocytes (del Santo et al., 1998, 2001; Dragan et al., 2000) being regulated by glucocorticoids and hepatocyte-specific transcription factors (Valdes et al., 2006; Fernandez-Veledo et al., 2007). On the other hand CNT3, which is not expressed in normal hepatocytes but shows broad expression in other epithelial tissues, such as colon and biliary epithelia, also appears to be a major player in regulating extracellular adenosine levels. In fact, CNT3 is under purinergic control via A2a adenosine receptors in cholangiocytes, thereby contributing to end up the initially driven purinergic control of bile flow started by ATP secretion into the bile (Godoy et al., 2014). This evidence suggest that selected NT proteins can indeed be part of the purinome, the molecular network of nucleoside and nucleotide receptors (P1 and P2), enzymes, and transporters responsible for purinergic regulation of cell functions (Volonte and D’Ambrosi, 2009). As long as some of the membrane proteins within the purinome, and other drug transporters as well, might eventually exert physiological effects other than the mere uptake or release of drugs, it can be anticipated that clinical implications of the changes in the transportome associated with the progression of disease will have to be evaluated from different perspectives and are likely to become suitable biomarkers and even drug targets.

## FUTURE PERSPECTIVES

Academic research focused on human NT proteins has established so far the basis for anticipating a probable role of these membrane proteins as biomarkers of diagnosis and prognosis. One major bottleneck in translational research within this field is the lack of suitable tools. Highly reliable antibodies are needed for both immunohistochemistry and flow cytometry, although quantitative proteomics can also prove to be suitable for biomarker analysis. This would enable more comprehensive, well structured, prospective clinical studies. The determination of NT protein expression during oncogenesis and the likely relationship between changes in the NT profile and stages of tumor progression would make these biomarkers very robust, although this progress is highly dependent upon the availability of well characterized clinical specimens. A major breakthrough within the field might come from the elucidation of NT interactomics, aiming at linking NTs with other cellular events that would help in the understanding of the biological basis of their use as biomarkers.

## Conflict of Interest Statement

The authors declare that the research was conducted in the absence of any commercial or financial relationships that could be construed as a potential conflict of interest.

## ABBREVIATIONS

CLL: chronic lymphocytic leukemia
CMV: cytomegalovirus
CNT: concentrative nucleoside transporter
DFS: disease free survival
ENT: equilibrative nucleoside transporter
HBV: hepatitis B virus
HCV: hepatitis C virus
HIV: human immunodeficiency virus
HSV: herpes simplex virus
NBTI: nitrobenzylthioinosine
NT: nucleoside transporter
OAT: organic anion transporter
OCT: organic cation transporter
OCTN: organic cation/carnitine transporters
OS: overall survival
PEPT: peptide transporter
SLC: solute carrier transporter
SVR: sustained virological response.
